# Metabonomic Analysis of Water Extracts from Different *Angelica* Roots by ^1^H-Nuclear Magnetic Resonance Spectroscopy

**DOI:** 10.3390/molecules19033460

**Published:** 2014-03-20

**Authors:** Pui Hei Chan, Wendy L. Zhang, Chung-Ho Lau, Chi Yuen Cheung, Hector C. Keun, Karl W. K. Tsim, Henry Lam

**Affiliations:** 1 Department of Chemical and Biomolecular Engineering, The Hong Kong University of Science and Technology, Clear Water Bay, Hong Kong, China; E-Mails: nichhan@ust.hk (P.H.C.); cycheungad@stu.ust.hk (C.Y.C.); 2 Divison of Life Science and Centre for Chinese Medicine, The Hong Kong University of Science and Technology, Clear Water Bay, Hong Kong, China; E-Mails: lizhang@ust.hk (W.L.Z.); botsim@ust.hk (K.W.K.T.); 3 Department of Surgery and Cancer, Faculty of Medicine, Imperial College London, Sir Alexander Fleming Building, South Kensington, London SW7 2AZ, UK; E-Mails: esmond.lau06@imperial.ac.uk (C.-H.L.); h.keun@imperial.ac.uk (H.C.K.); 4 Division of Biomedical Engineering, The Hong Kong University of Science and Technology, Clear Water Bay, Hong Kong, China

**Keywords:** *Angelica sinensis*, *Angelica gigas*, Traditional Chinese Medicine, NMR, metabonomics, metabolomics

## Abstract

Angelica Radix, the roots of the genus *Angelica*, has been used for more than 2,000 years as a traditional medicine in Eastern Asia. The Chinese Pharmacopoeia records more than 100 herbal formulae containing *Angelica* roots. There are two common sources of *Angelica* roots, *Angelica sinensis* from China and *A. gigas* from Korea. The two species of *Angelica* roots differ in their chemical compositions, pharmacological properties and clinical efficacy. ^1^H-NMR metabolic profiling has recently emerged as a promising quality control method for food and herbal chemistry. We explored the use of ^1^H-NMR metabolic profiling for the quality control of Angelica Radix. Unlike previous work, we performed the metabolic profiling on hot water extracts, so as to mimic the clinically relevant preparation method. Unsupervised principle component analyses of both the full spectral profile and a selection of targeted molecules revealed a clear differentiation of three types of *Angelica* roots. In addition, the levels of 13 common metabolites were measured. Statistically significant differences in the levels of glucose, fructose and threonine were found between different sources of *Angelica*. Ferulic acid, a marker commonly used to evaluate *Angelica* root, was detected in our samples, but the difference in ferulic acid levels between the samples was not statistically significant. Overall, we successfully applied ^1^H-NMR metabolic profiling with water extraction to discriminate all three sources of *Angelica* roots, and obtained quantitative information of many common metabolites.

## 1. Introduction

Angelica Radix, the roots of the genus *Angelica* (“Danggui” in Chinese), has been used for more than 2,000 years as a traditional medicine in China, Korea and Japan. Angelica Radix is often called “female ginseng” because it is traditionally applied to the treatment of gynecological disorders [1]. The medical use of *Angelica* root was first recorded in ~100 B.C in *Shen Nong Bencao Jing*, which stated that *Angelica* root is effective in replenishing the blood, reducing pain and moistening the intestine. Clinically, Angelica Radix can be applied to treat anemia [2], to enhance the immune system [3] and to relieve constipation [4]. The Pharmacopoeia of People’s Republic of China (PRC) records more than 100 herbal formulas containing *Angelica* roots in China and Japan [5]. Nowadays, *Angelica* root is not only commonly consumed in Asia, but also in the Western countries as a health food supplement. The Chinese Pharmacopoeia (2010) defines that *Angelica* root in China is derived from the root of *Angelica sinensis* (Oliv.) Diels. However, *Angelica* *acutiloba* (Sieb. *et* Zucc.) Kitag., mainly found in Japan, and *Angelica* *gigas* Nakai, mainly found in Korea, are commonly used instead of *A. sinensis* in Japan and Korea. The three commonly used *Angelica* roots showed variation in their compositions, pharmacological properties and efficacy [6]. In addition, the cultivated regions of *Angelica* also have a significant difference in chemical quantities and biological response [7] even when they are parts of a decoction [8]. The study concluded that the source of herb should be considered when preparing decoctions in order to achieve the maximum biological efficacy and minimum toxicity [8]. Thus, the authentication of different *Angelica* species is an important task.

*Angelica* species were previously distinguished by sequencing of internal transcribed spacers, nuclear ribosomal DNA, 5S-rRNA spacer and 18S-rRNA [6]. Although these DNA-based methods are effective, they can only be applied to the crude herb and not to the prepared product, such as ready-to-consume decoctions or tablets. Chemical marker identification is also applied on the quality control of *Angelica*; ferulic acid and *Z*-ligustilide were chosen as the markers for the quality control [5,9]. These two compounds however are not unique to *Angelica*, and are not necessarily clinically relevant. It is also unclear if these markers are sufficient to distinguish the closely related *Angelica* species. 

Recently, there is an increasing interest in using metabolic profiling approaches to obtain comprehensive chemical signatures for quality control of herbal medicines [10,11,12]. Kim *et al.* have applied ^1^H-NMR and UPLC-MS for the quality control of *A.* *gigas* of different geographical origins in Korea [13]. By using methanol extraction with UPLC-MS, they successfully discriminate *A. gigas* from 3 different regions in Korea. Kobayashi *et al.* applied GC-MS metabolic profiling to discriminate *A. acutiloba* and *A. sinensis* [11]. Here, we demonstrated that ^1^H-NMR metabolic profiling could be used to distinguish the hot water extracts of two different species of Angelica Radix from three geographically different cultivation regions. We established that mainly by measuring primary metabolites, ^1^H-NMR metabolic profiling can be used to distinguish these closely related herbal samples, even if a less potent extraction method is used. Importantly, by employing a more clinically relevant method of preparation of boiling in water, instead of methanol extraction used in previous studies, we showed that our method could be applied to commercial ready-to-consume herbal products.

## 2. Results and Discussion

**Figure 1:**
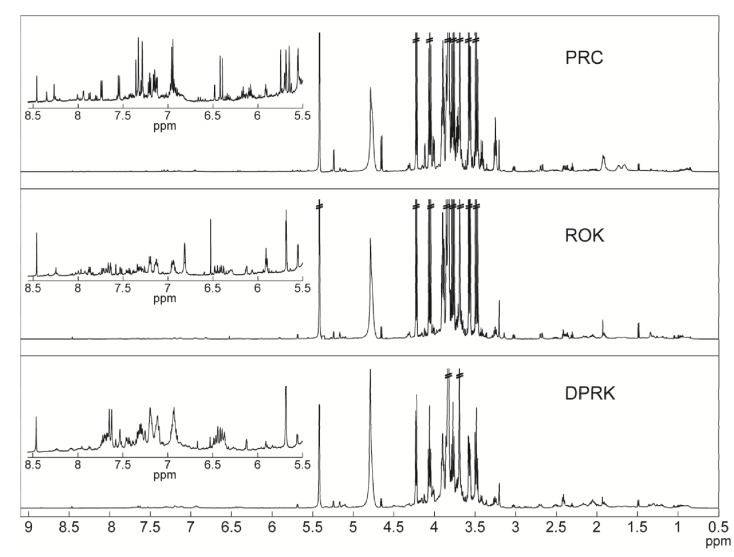
Average ^1^H-NMR Spectra of three *Angelica* extracts, *A. sinensis* from China (PRC) and *A. gigas* from ROK and DPRK. Spectra represented the mean of five replicates. By visual inspection, the average ^1^H-NMR spectra of the three herbs showed a very similar profile, but observable differences in the finer details. The most intense peaks were found between 3.3–4 ppm, which are associated with carbohydrates. A visible difference in peak pattern was also found between 5.5–8.5 ppm, associated with aromatic compounds.

The average spectra of *A. sinensis* from China and *A. gigas* from ROK and DPRK were obtained and are shown in Figure 1. Visual inspection showed that all three spectra were indeed rather similar. The most intense peaks were found between 3.3–4 ppm, which corresponded to sugar compounds. Sucrose was the highest concentration among these peaks, in good agreement with the result of Kim *et al*. [13], who employed methanol extraction. In addition, we identified glucose and fructose, as well as other primary metabolites including a few amino acids and organic acids. However, we could not detect the specific secondary metabolites, such as decursin and other coumarin derivatives, as revealed by Kim *et al.* [13]. Decursin is considered as one of the active compounds in *Angelica*, especially in *A. gigas*, which is reported to have beneficial effects against cancer, oxidative stress, neurological disorders, fat accumulation and obesity-induced diabetes [14]. Comparing our results to Kim *et al.*, we concluded that the typical method of preparing *Angelica* roots for consumption (*i.e*., boiling in water), which we mimicked in this study, did not produce detectable levels of these useful compounds. 

**Figure 2:**
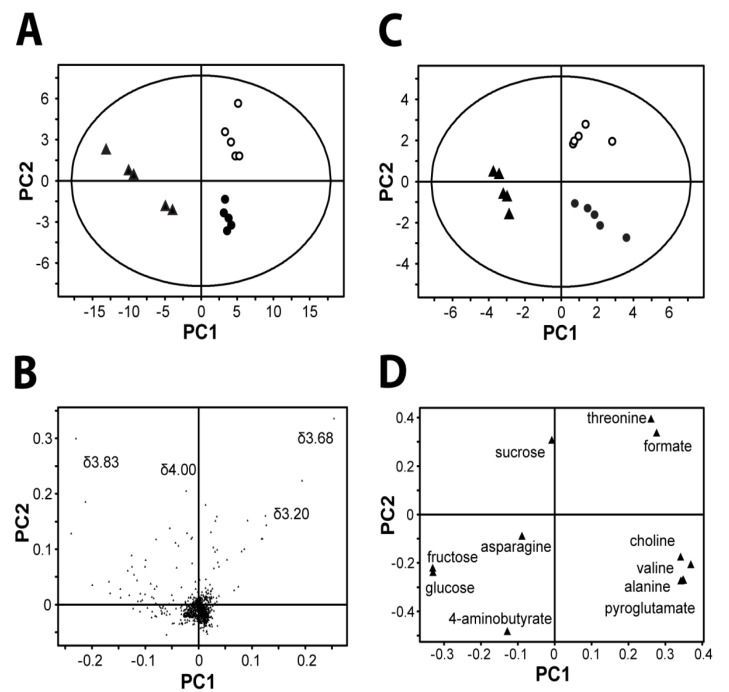
Chemometric analysis by principle component analysis (PCA). (**A**) and (**B**): Score and loading plots from pattern recognition (PCA) of whole ^1^H-NMR spectra are shown. The data sets are *Pareto*-scaled. Overall, discrimination of all three groups of *Angelica* (*n* = 5) was clearly shown in the score plot. The loading plot shows that 3.2–4 ppm, the sugar region, is the most discriminating feature. (PC1: R2 = 0.70, Q2 = 0.63; PC2: R2 = 0.86, Q2 = 0.78) (**C**) and (**D**): Scores and loading plots from pattern recognition (PCA) of a selection of targeted molecules. Data are unit-variance scaled. Loading plot shows which molecules contributed most to the discrimination. (PC1: R2 = 0.56, Q2 = 0.34; PC2: R2 = 0.74, Q2 = 0.36). Abbreviation: PRC, *A. sinensis* from China; DPRK, *A. gigas* from Democratic People’s Republic of Korea; ROK, *A. gigas* from Republic of Korea.

Reduced-resolution spectra (~1,100 data points representing integrated regions of 0.01 ppm width) were exported to SIMCA-P for multivariate analysis. Principle component analysis (PCA) was applied to data analysis. PCA is the most basic and efficient method for analyzing complex data in metabonomics, which helps to extract and display systematic variations from the data, as well as detects groupings, trends and outliers if they are present in the data [15]. Figure 2A shows a PCA plot. Each point in the PCA score plot represented a single sample, and samples clustered together were considered to have similar characteristics—in this case, similar metabolic profiles. The three *Angelica* roots, *A. sinensis* root (China) and *A. gigas* roots (ROK and DPRK) were successfully discriminated based on their water extracts alone. By studying the corresponding PCA loading plots, the discriminating features were clearly identified (Figure 2B). The most discriminating peaks are found in the sugar region between 3.4–4 ppm. In order to understand how other metabolites also contribute to the discrimination, we then performed PCA on the integrated peak areas of the identified metabolites only (Figure 2C). Clear separation of the three *Angelica* samples was similarly obtained. The corresponding loading plot indicated that *A. sinesis* from China showed a higher concentration of glucose, fructose and 4-aminobutyrate, while threonine, formate and succinate were found higher in *A. gigas* from ROK. Choline, valine, acetate, alanine and pyroglutamate were found at higher levels in *A. gigas* from DPRK (Figure 2D).

**Figure 3:**
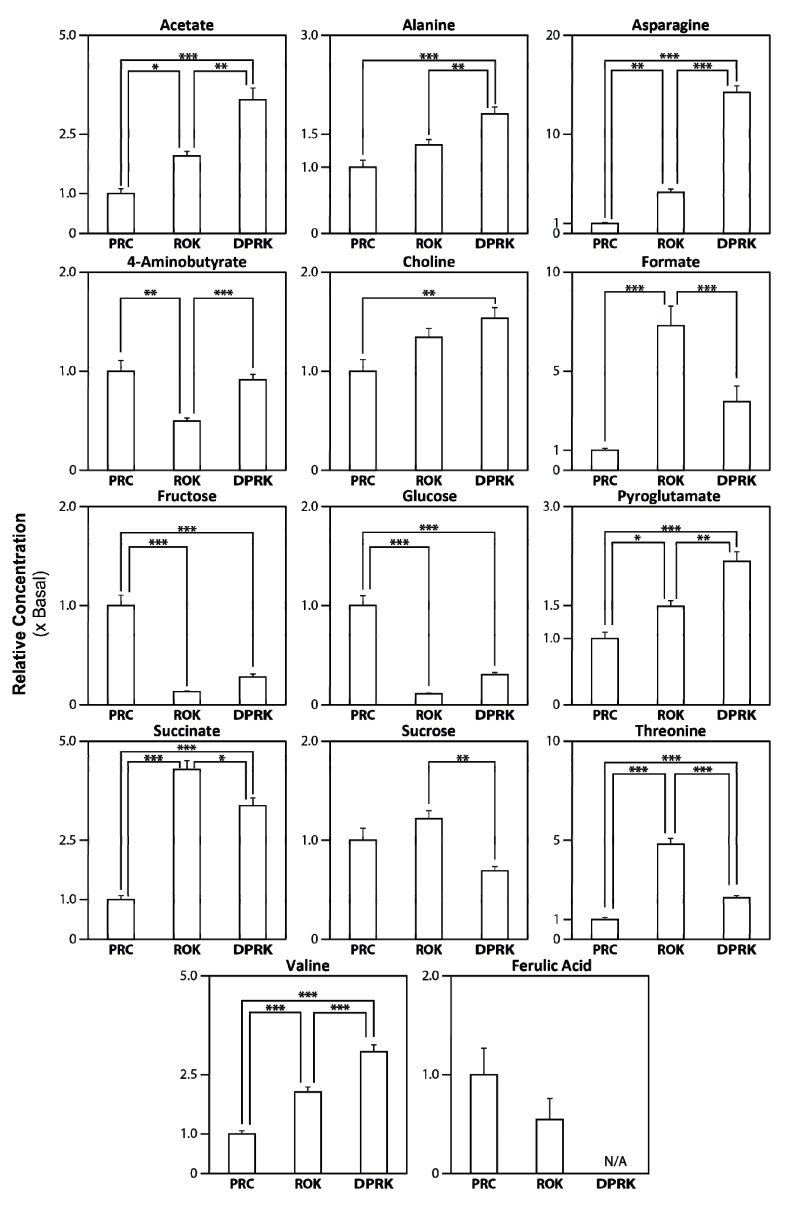
Quantification of identified metabolites in the different *Angelica* extracts. The peaks associated with identified metabolites (listed in Table 1 with respective chemical shifts) in all of the ^1^H-NMR spectra were integrated to yield the concentrations in the extracts relative to the mean value for *A. sinensis* (PRC) (basal level set at 1.0). The values are expressed as Means ± SEM (*n* = 5). * *p* ≤ 0.05, ** *p* ≤ 0.01, *** *p* ≤ 0.001, by one-way ANOVA followed by post-hoc multiple comparison tests. Abbreviations: PRC, *A. sinensis* from China; DPRK, *A. gigas* from Democratic People’s Republic of Korea; ROK, *A. gigas* from Republic of Korea.

All identified and integrated peaks were further evaluated with Student t-tests (Figure 3 and Table 1). Sucrose was found at the highest intensity in *Angelica* roots, followed by fructose and glucose. One-way ANOVA determined that the two *A. gigas* groups (ROK and DPRK) have significantly lower (*p* < 0.001) levels of glucose and fructose than the *A. sinensis* (PRC) group, and those sugars alone cannot distinguish the two *A. gigas* groups significantly. Among the other metabolite examined, several amino acids, including threonine, asparagine and valine, can alone differentiate all three groups (*p* < 0.01). Acetate, alanine, asparagines, choline, pyroglutamate and valine exhibit similar trends, with highest levels found in *A. gigas* from DPRK, followed by *A. gigas* from ROK (Table 1 and Figure 3). Levels of these metabolites are lowest in *A. sinensis*. 

**▲** *A. sinensis* (PRC) **●** *A. gigas* (DPRK)** ○** *A. gigas* (ROK)

**Table 1:** Chemical shifts of metabolites identified in ^1^H-NMR Spectra of different sources of *Angelica* roots used for quantification.

Metabolite	Chemical Shift (ppm)
Acetate	1.9(s)
Alanine	1.5(d)
Asparagine	2.93 (m)
4-aminobutyrate	2.3(t)
Choline	3.2(s)
Formate	8.4 (s)
Fructose	4.6(d)
Glucose	5.2(d)
Pyroglutamate	2.0(m)
Succinate	2.4(s)
Sucrose	5.4(d)
Threonine	1.3(d)
Valine	1.1(d)
Ferulic Acid	6.3(d)

Ferulic acid (δ 6.3) and *Z*-ligustilide are often considered as important biomarkers in *Angelica*. Ferulic acid inhibits platelet aggregation and serotonin release while *Z*-ligustilide has anti-asthmatic and spasmolytic activity [1]. By simple solubility tests using pure standards, we found that *Z*-lingustilide is water insoluble, while ferulic acid has a low water solubility (~6 mg/mL). Kim *et al.* did not show that they could detect Z-ligustilide or ferulic acid by methanol extraction with ^1^H-NMR and UPLC-MS [13]. Similarly, Kobayashi *et al.* did not report observing these two markers by GC-MS metabolic profiling on *A. acutiloba* and *A. sinensis* [11]. Here, *Z*-ligustilide was not detected in the ^1^H-NMR spectra, but ferulic acid was observed at 6.3 ppm. Our result suggests that *A. sinensis* from China has a higher average ferulic acid concentration than *A. gigas* from Korea but the difference is not statistically significant. This result partially agreed with Zhao *et al.* [6], who also detected higher ferulic acid concentrations in *A. sinensis* than in *A. gigas*. However, we did not observe as large as difference as that was reported by Zhao *et al.* [6]. The level of ferulic acid in the DPRK *A. gigas* sample was not detected, because the peak of interest at 6.3 ppm was overlapped with another substantial peak of unknown identity. Unresolved peaks in our analyses could potentially be identified and quantified by using 2D-NMR or hyphenated techniques, such as LC-NMR, in the future. 

We have applied NMR metabolic profiling as our tool for the quality control of *Angelica* roots. We detected many common amino acids and sugars, instead of the presumed active ingredients of *Angelica*. This could be due to the water extraction that we used here instead of methanol extraction used in previous studies. These active compounds might be too hydrophobic to be extracted efficiently by boiling water, resulting in a concentration lower than the detection limit of our ^1^H-NMR method (approximately 10 μM). Although these compounds were not detected, we still managed to distinguish the different sources of *Angelica* roots, based on their primary metabolites.

NMR profiling is a promising method for quality control of commercial herbal medicine products, with its quick analysis time (about 5 min per sample), and its simple sample preparation. It is also unbiased (all compounds with protons are profiled) and non-destructive. Although ^1^H-NMR spectra of mixtures may exhibit overlapping peaks, which poses challenges in peak identification and integration, this does not interfere with the reproducibility of ^1^H-NMR for the purpose of sample differentiation and quality control. In comparison, LC-MS metabolic profiling has a higher sensitivity than ^1^H-NMR (10^−9^–10^−11^ mol in comparison with 10^−19^ mol), and which can detect lower-abundance metabolites [16]. However, absolute quantification is impossible with LC-MS, unless pure standards are available, and the experimental and data analysis procedure of LC-MS is usually more time-consuming. 

## 3. Experimental

### 3.1. Materials

Fresh plants were obtained from China: *A. sinensis* from Minxian, Gansu were collected by the authors; *A. gigas* from Chuncheon, Republic of Korea (ROK) and Democratic People’s Republic of Korea (DPRK) were collected by Dr. H. Xiu of National Product Chemistry Laboratory, Department of Applied Biological and Environmental Chemistry, Seoul National University. The plant materials were collected in September to October after they had been cultivated for two years. The voucher specimens were deposited in the Centre for Chinese Medicine R&D at the Hong Kong University of Science and Technology. 

### 3.2. Preparation of Decoctions

About 15 g of dried, sliced *Angelica* root from different sources was weighed, boiled in 120 mL of water for 2 h, and extracted twice. For the second extraction of *Angelica* roots, the residue from the first extraction was filtered, and the same extracting conditions were applied. The extract was dried under vacuum and stored at −80 °C for further analysis. The extraction process was repeated 5 independent times for different batches of *Angelica* roots, as characterized in this study.

### 3.3. Sample Preparation for NMR Spectroscopy

Fifty mg of the dried extract was dissolved in 1 mL of water, from which 550 μL was mixed with 50 μL of a D_2_O solution with 0.2% TSP-d4 and 3 mM sodium azide. TSP-d4 acted as an internal frequency reference for NMR, and sodium azide inhibited microbial growth. The mixture was centrifuged at 13,000 ×*g* for 1 min, and the supernatant was transferred to a standard 5-mm NMR tube. NMR spectra were acquired on a Bruker AV 400MHz NMR spectrometer with a 5-mm PA BBO 400SB BBFO-H-D05 Z-gradient BB observed probe head, operating at 400.13 MHz ^1^H-NMR frequency at 298 K. Gradient shimming was used to improve the magnetic field homogeneity prior to all acquisition. ^1^H-NMR spectra of the samples were acquired using a 1D NOESY pulse sequence (RD-90°-t_1_-90°-t_m_-90°-acquire) applying presaturation during recycle delay (RD, 2s) and mixing time (t_m_, 50 ms) to generate a spectrum with a reduced residual solvent peak. The experiment time for each sample was around 5 min (64 scans). All spectra were Fourier-transformed, phase-corrected and baseline-corrected manually.

### 3.4. Data Analysis

^1^H-NMR spectra obtained from each sample were calibrated to the TSP-d4 internal standard resonance (δ_H_ = 0.00), normalized to the integrated peak area of the TSP resonance, and imported into MATLAB (release 2009b, MathWorks, Natick, MA, USA) using a proprietary script written by Rachel Cavill, Hector Keun, and Tim Ebbels (Imperial College, London, UK). Spline interpolation was employed to produce NMR spectra of 32,000 data points in the frequency domain between −1 and 10 ppm. Reduced-resolution spectra (~1,100 data points representing integrated regions of 0.01 ppm width) were imported into SIMCA-P version 12.0 (Umetrics, Umeå, Sweden) for principle component analysis (PCA). Prior to PCA, the data were *Pareto* centered and each variable scaled to unit variance. The resulting PCA model was used to pinpoint the peaks in spectra that were different between different groups. These were then assigned using Chenomx Profiler, a module of Chenomx NMR Suite version 7.5, and additional information from the online databases [17,18] and a review [19]. The statistical significance of the integrated area of these assigned peaks was tested using one-way ANOVA, followed by post-hoc multiple comparison tests by Tukey’s HSD method. Metabolite concentrations were estimated by calibration of the integrated peak area to the TSP resonance and accounting for proton numbers contributing to each resonance.

## 4. Conclusions

By using water extraction with ^1^H-NMR metabolic profiling techniques, we successfully discriminated *A. sinensis* from China and *A. gigas* from ROK and DPRK. Importantly, our method of boiling *Angelica* root in water mimics the typical method used to prepare the herb for consumption. In this study, we noticed that some of the chemicals reported to offer benefits to humans, such as decursin and *Z*-liguistilide, could not be detected in the water extracts by ^1^H-NMR. This is likely because the levels of these molecules in the water extracts are below the detection limit of NMR (~10 μM). This also suggests that previous studies that employ methanol extraction to determine the abundance of these compounds might not tell an accurate story of how much they are actually consumed, or which *Angelica* species are more beneficial to humans.
